# An Experimental Investigation of Static Properties of Bio-Oils and SAE40 Oil in Journal Bearing Applications

**DOI:** 10.3390/ma15062247

**Published:** 2022-03-18

**Authors:** Muhammad Imran Sadiq, Wan Aizon Ghopa, Mohd Zaki Nuawi, Mohammad Rasidi Rasani, Sofian Ibrahim

**Affiliations:** 1 Department of Mechanical and Manufacturing Engineering, University Kebangsaan Malaysia, Bangi 43600, Selangor, Malaysia; waizon@ukm.edu.my (W.A.G.); mzn@ukm.edu.my (M.Z.N.); rasidi@ukm.edu.my (M.R.R.); 2 Malaysian Nuclear Agency, Kajang 43000, Selangor, Malaysia; sofian_ibrahim@nm.gov.my

**Keywords:** journal bearing, rotor dynamics, bio-lubricants, dynamic viscosity, modal analysis

## Abstract

Considerable research has been conducted in the past decade and a half regarding the bio-lubricants potential to replace mineral-based lubricants as mainstream lubricants such as engine oil, hydraulic oil, compressor oil, and metalworking oil. This study studied several bio-lubricants (rapeseed oil, palm olein, and soybean oil) and a mineral-based lubricant, SAE40. The bio-lubricants have better physiochemical, tribological characteristics and environmental friendly nature, and are promising to replace mineral-based lubricants. In this study, a journal bearing test rig (JBTR) was developed in order to investigate the effect of journal speed on the temperature of oil film with time. Additionally, the load-carrying capacity of bio-oils was tested against the mineral-based lubricant SAE40 by adding a load on the journal. For all three speeds, i.e., 1000, 1500, and 2000 rpm, the bio-lubricants recorded minimum temperature. At 1000 rpm, rapeseed oil recorded a 9.2% lower temperature than SAE40. Similarly, at 2000 rpm, rapeseed oil recorded a minimum temperature that was 2.5% lower than SAE40; in comparison, at 1500 rpm, palm olein recorded a minimum temperature that was 1.8% less than SAE40. Overall, the results of this study revealed that bio-oils recorded a lower temperature rise than mineral oil. These results are very encouraging for further research in this area.

## 1. Introduction

With the increase in the world’s population, combined with industrialization, there has been a rise in the use of mineral-based oil resources and fossil fuels. These resources are nonrenewable and are also quickly depleting. It is very important to introduce new alternatives using renewable energy sources that help reduce the burden on these fossil fuel resources and also protect our mother nature. Bio-oils, which are extracted from natural renewable resources, are not toxic to the environment. They display excellent physicochemical properties, such as high viscosity index and flash point, as well as good resistance to shear and high biodegradability. Lubricants, with the help of certain additives, perform multiple tasks in rotating machinery. These are used to carry out heat transfer, provide a damping effect, and carry the loads by the formation of the oil film in journal bearings. In a rotor-bearing system, lubricants play very significant roles in defining the tribological and dynamic characteristics of the system. 

There are three different types of lubrication, classified as the boundary, mixed, and full-film types. Each type is different, but they all constitute a lubricant and the additives within the oils to protect against wear and friction. Full-film lubrication can be broken down into two forms—hydrodynamic and elastohydrodynamic. Additionally, the lubricants are generally liquids. However, they are often used in solid (graphite), semi-solid (greases), and gaseous forms.

Viscosity is one of the most important properties of a fluid lubricant that determines the fluid friction involved in lubrication, the load-carrying capacity of the lubricant film, its resistance to the initiation of relative movement of moving parts and the sealing capacity, pumping ability, and heat transfer properties of the lubricant. It is a measure of the internal friction occurring in a fluid, which is the mutual resistance to the relative motion of the fluid molecules. If the viscosity of a liquid is not constant at a given temperature and pressure but depends on shear rate, its viscosity is termed apparent viscosity, and the liquid is a non-Newtonian fluid. It is reported that the fluid properties of such a substance must be measured using a variable shear-rate viscometer [1].

It is very important to consider the operating temperature for lubricant when choosing a lubricant for the desired operation [2]. In many lubricants, properties such as temperature and pressure are somewhat comparable, but the shear response is different. It is found that above certain shear stress levels, different lubricants begin to exhibit nonlinear behavior. This value of shear stress varies for different types and classes of lubricants. Therefore, it is an indication that the lubricant has started to behave as a non-Newtonian lubricant. This does not primarily mean that the lubricant is non-Newtonian, rather the operating conditions have caused this behavior [3]. Similarly, in plain journal bearings lubricated by water, seawater, and oil, the maximum temperature occurs at the minimum film thickness location, although it extends 180° backward for water and seawater. Additionally, for oil-lubricated bearing, the thermal effect has a greater effect on pressure distribution than does elastic deformation [4]. Mineral-based oils have been widely used as the main lubricant oil. However, these lubricants are toxic, nonbiodegradable, and not environmentally friendly. Mineral-based lubricants contain many kinds of additives such as antioxidants, anti-wear, detergents, dispersants, antifoams, extreme pressure agents, friction modifiers, and viscosity enhancers. Some of these additives are toxic and harmful to human health, wildlife, and the environment [5,6]. Due to environmental concerns, research toward finding alternative oils for lubricants has currently become very demanding.

Rubbing is a phenomenon that generally occurs in journal bearings. Many efforts have been made to reduce the rubbing phenomenon. One of the ways is by adding nanoparticles to the lubricant. It subsequently reduces rubbing and improves the tribological properties of lubricants in journal bearing [7]. A bio-lubricant is a renewable lubricant that is biodegradable, nontoxic, and emits net-zero greenhouse gas. Bio-lubricants have been found to exhibit superior lubricant tribological properties over conventional mineral lubricants, with renewability and biodegradability being their strongest point. In general, bio-lubricants (pure and chemically modified) have been reported to have good tribological properties, compared with mineral-based lubricants. The improved tribological performance of bio-oils is due to the fact that high oleic acid concentrations improve friction and wear performance by establishing densely packed monolayers on the lubricating surface. Moreover, the benefits of bio-oils include lower pollution (air, water, and soil), minimal health and safety risks, and easier disposal due to their facile biodegradability [8,9]. In comparison to mineral-based lubricants, bio-lubricants have good tribological properties, can perform better when properly formulated and processed with additives. Additionally, it is reported that bio-oils exhibit low volatile organic compound emission for engine oil application, low compressibility and fast air release rate for the hydraulic application, and high dielectric strength for transformer application [10]. Furthermore, it is also reported that blending of bio-oils with mineral-based lubricants up to a certain level yields optimum results in terms of coefficient of friction (COF), viscosity, rise in temperature and wear scar diameter (WSD), and load-carrying capacity [11,12]. Anti-wear coatings are also used to achieve better thermal response [13]. Similarly, the addition of boric acid into vegetable oils leads to an increase in the lubrication efficiency by up to 15%, compared with pure oils [14].

Chemical modification of bio-oils to chemically modified bio-based lubricant is a process in which the properties of bio-oils are optimized as per the requirements and needs of the industry. Structurally and chemically modified bio-based lubricants have exhibited improved viscosity and good thermo-oxidative stability [15]. Blending the nanoparticles with the base lubricant does not change the rheology of lubricants. The addition of nanoparticles (0.5%) to chemically modified bio-based lubricants has been reported to have improved viscosity and increased flash point, leading to better lubricity, and less wear and damage to the surface due to the formation of a better film on the surface [16]. Tests carried out on the pin with a disc wear tester show that vegetable oil can be used as a potential bio-lubricant and an alternative to conventional industrial lubricants; nevertheless, there is still a need for more research to be carried out [17,18]. It is further reported that slight modification in physical properties and the use of additives can cause a rise in the use of bio-lubricants in biomedical applications [19]. Similarly, it is important to consider the economic and environmental aspects along with other parameters. Good physiochemical and tribological properties of bio-oils can contribute to the reduction in energy loss from friction, improve fuel economy, reduce greenhouse gas emissions, and generate economic savings; therefore, they are beneficial in terms of both economic and environmental aspects [20].

Good lubricating properties, high viscosity index, high ignition temperature, increased equipment service life, high load-carrying abilities, good anti-wear characteristics, excellent coefficient of friction, natural multigrade properties, low evaporation rates, low emissions into the atmosphere, and rapid biodegradability are some of the highlight features which make the bio-lubricants attractive, compared with mineral-based lubricants [21,22]. Optimization of lubricants/oils, keeping in mind the industry in which the bearings are used, is of prime importance. The overall power loss of the machine can be reduced by using an oil with better kinematic viscosities because, at higher speeds of rotation, the viscosity of the lubricant plays a very important role in defining the overall trends of vibration response. The optimized dynamic viscosity curve is obtained and can be used for the formulation of a type of oil, not necessarily of mineral origin, but with suitable additives [23]. Similarly, depending upon the use, the bio-lubricants are reported to be used as base stock, in mixtures with mineral-based lubricants, and as blended with additives. Bio-lubricants have poor cold flow properties and low oxidation, but these shortcomings can be addressed by modifying the bio-lubricants chemically or incorporating additives [24,25]. 

Therefore, it is very important to experimentally test these bio-oils in actual operating conditions for the evaluation of static and dynamic performance. In this study, a setup was developed to test the performance of bio-oils, after which the performances were compared with those of the mineral-based lubricant. Hence, in this paper, rapeseed oil, palm oil, and soybean oil were tested and compared with SAE40, a mineral-based oil, to evaluate their performance in JBTR under similar operating conditions. The three bio-oils used in this study were in unprocessed and untreated form.

## 2. Materials and Methods

### 2.1. Design of Journal Bearing Test Rig (JBTR)

It is very important to design a test rig to simulate the operating conditions and to be able to record the measurement data with repeatability and accuracy [26,27]. It is likely that if different operators are using the same equipment, the results might vary depending upon the level of experience and sensitivity to equipment. The standard operating procedures may be prepared beforehand to avoid any such problems [28]. It is essential to develop a system that can easily be tested for different lubricants with additives. Furthermore, to make provision for sensors at axial and radial locations, vibration analysis is also needed for testing and analysis purposes. The test rig needs to be flexible in order to deal with shafts of large diameters and high speeds. Moreover, there should be provision for modernization and further development of test rig [29]. Furthermore, many bearings work in flooded conditions with more than the minimum required oil [30]. Therefore, it is important to make sure that the bearing has a sufficient supply of oil in order to avoid friction and rubbing and obtain more accurate results.

**Figure 1:**
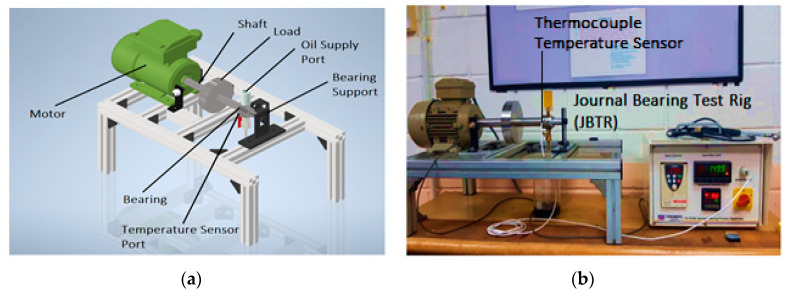
(**a**) Schematic diagram; (**b**) actual diagram of journal bearing test rig (JBTR).

For this purpose, a journal bearing test rig (JBTR) was developed to experimentally validate the physical properties of the bio-oils and compare them with SAE40. The model and real system can be seen in Figure 1. The oil was supplied to the bearing through the oil supply port, which was at the top of the journal bearing. It could contain up to 15 mL of oil. Provision was made for recirculation, refilling, and replacement of oil depending upon the requirement. A DC motor equipped with a speed control unit that can operate up to 3000 rpm (50 Hz) was directly attached to the journal. A tachometer was placed near the shaft to record the speed of the shaft in revolution per minute (RPM). The load was applied to the journal bearing through a disk mounted on the journal and could be changed as per loading conditions. Provision was made for temperature measurement in this test rig by using a thermocouple sensor. The main control unit set the desired rpm, recorded the actual rpm of the shaft, and recorded the temperature of the oil. The specifications of the journal bearing test rig are given in Table 1.

Figure 1a,b show the schematic model and the actual JBTR system. This system can be used to test different types of oils without any complex mechanism, as the oil can be easily replaced. Additionally, the system has the ability to test the load-carrying capacity for different types of loads at different rotating speeds.

### 2.2. Experimental Modal Analysis

The modal analysis was carried out to determine the natural frequencies and associated mode shapes to ensure our test rig is safe to operate in the given range. It is important to investigate the natural frequencies in order to investigate whether the resonance would occur in the operating range. Additionally, how the system responds at those natural frequencies is also important to investigate. During the design process, it is important to make sure that there is no natural frequency occurring within the operational range of the rotating machine unless it is a design requirement [31]. Otherwise, the system may experience resonance, resulting in high vibration, which may lead to failure of the system.

For experimental modal analysis, the impact testing technique was used, as this is more suitable for this kind of system. This test was carried out in accordance with ISO 7626-5 standard. A modally tuned impact hammer was used for excitation, and the response was measured using a triaxial accelerometer PCB 356A01. The accelerometer was connected to a data acquisition and analysis system, Rionote SA-A1.

### 2.3. Numerical Modal Analysis

**Figure 2:**
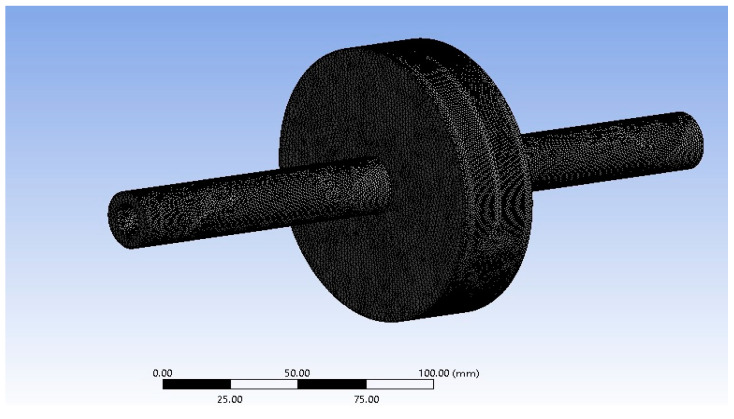
Numerical modal analysis.

Numerical modal analysis was performed using the commercial software ANSYS workbench R2 2021, as shown in Figure 2. For boundary conditions, there was fixed support at one end and elastic support at the other end. Material properties for modal properties are listed in Table 2.

Figure 2 shows the numerical model which is developed to determine the natural frequencies of the system. This system comprised the basic components that included shaft and load because only the shaft and the load were the rotating components, and the remaining components of the whole system were stationary.

### 2.4. Dynamic Viscosity Measurement

Dynamic viscosity measurement was carried out in order to determine the response of lubricants with increasing temperature. Temperature–viscosity characteristics of lubricating oils and greases are among the important parameters for evaluating lubricant performance in mechanical systems. The sustainability of lubricating film between contacting surfaces in mechanical systems is often criticized due to the highly sensitive nature of temperature–viscosity relationship in lubricants. Oil viscosity often decreases rapidly with a rise in temperature. Loss in lubricants viscosity may cause severe problems in terms of the performance of mechanical systems in industrial and transportation applications. 

**Figure 3:**
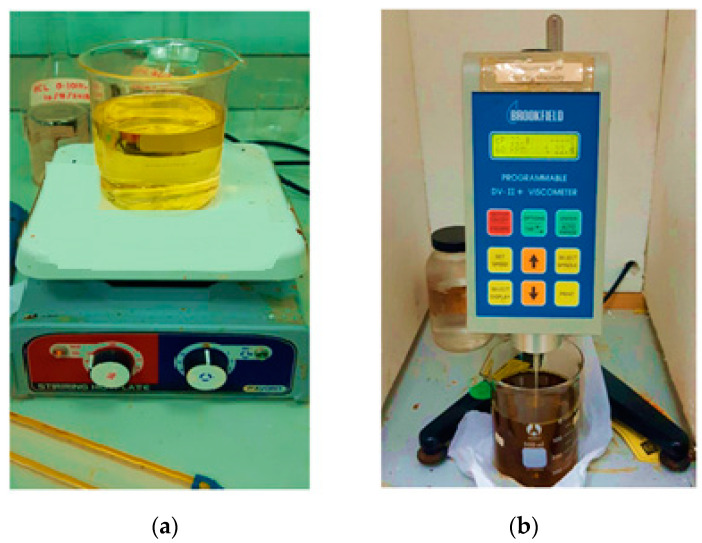
(**a**) Viscosity measurement. (**b**) The Brookfield Viscometer.

Dynamic viscosity is the measure of the shear stress per unit area required before a sample begins to deform. Lubricants are characterized by their viscosity as a function of temperature, pressure, and shear rates. However, viscosity is largely influenced by the working temperature of the liquid [32]. Viscosity is generally expressed in millipascal seconds (mPa-s) or centipoise (cP). Fluid viscosity changes with temperature. The increase in temperature causes a decrease in fluid viscosity. The viscosity of a liquid is measured using a device called a viscometer. In order to test the oil samples’ viscosity, the Brookfield Viscometer was used, as shown in Figure 3b. Figure 3a demonstrates the testing process, during which the oil was heated using the hot plate up to the desired temperature (40°, 50°, 100°, and 125 °C). Next, as shown in Figure 3b, the viscosity of the oil was checked at the given temperature. Different types of spindles were used to measure the viscosity for different ranges of viscosity. The spindle was attached to the apparatus, and it was made to rotate at 60 revolutions per minute (RPM). The viscosity was measured for different temperatures in order to investigate the effect of temperature on viscosity.

Figure 3a shows the oil sample being heated on the hot palte. Whereas, Figure 3b shows the viscometer, which is used for measuring the oil viscosity. Table 3 shows some important properties of the three bio-oils and SAE40 oil. The three bio-oils had almost similar flash points, which were almost 90 °C higher than that of SAE40. This is also an important factor given that the operational range of machines is at higher temperatures, for instance, 150~250 °C. Similarly, the density of bio-oils was greater than that of the SAE40 oil. Similar properties are also reported by [33].

### 2.5. Bearing Temperature Measurement

To investigate the effect of speed and load on the performance of given oil samples, a JBTR (Figure 1) was made to run for 60 min (1 h) in order to observe how the temperature increased at a given speed of the journal. Each oil sample was tested for three speeds of 1000, 1500, and 2000 rpm. The temperature measurement was carried out after 5 min, up to 60 min. Omron temperature controller E5CN was used to measure the temperature. This controller uses the thermocouple sensor to measure the temperature.

Thus, by carrying out the above-described procedures, the main hypothesis was to obtain actual experimental results and investigate how the bio-oils behave relative to temperature rise when put to test under operating conditions. Some of the tests such as viscosity measurement and other lubricity tests can provide insight into the behavior of bio-oils, but an actual experimental setup should be used to verify the hypothesis of having better static and dynamic characteristics.

## 3. Results and Discussion

**Figure 4:**
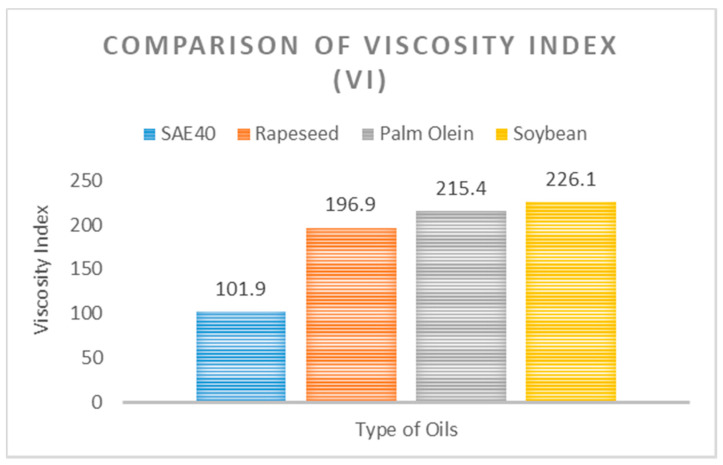
Viscosity index comparison.

In tribological systems such as gears, bearings, and engines, the temperature rise due to friction-induced heating is inevitable. Another major source of heat is the high-temperature operating environment, similar to that of steam and gas turbines. Therefore, it is important to introduce lubricants that have less viscosity loss and can maintain high viscosity, compared with the existing lubricants. Figure 4 shows the results of a comparison of viscosity index (VI) between bio-oils and SAE40.

In Figure 4, VI of bio-oils and SAE40 is plotted. The VI is unitless and indicates the change in fluid viscosity when measured against a change in temperature. A lower value of VI indicates that viscosity is affected more by the change in temperature and vice versa. Two values of temperature were considered to measure the VI, 40° and 100 °C. From Figure 4, it can be seen that the three bio-oils showed better VI, which was double or even higher for bio-oils, compared with the SAE40 oil. The graph in Figure 4 shows the dynamic viscosity trend in bio-oils and SAE40 at various temperatures.

**Figure 5:**
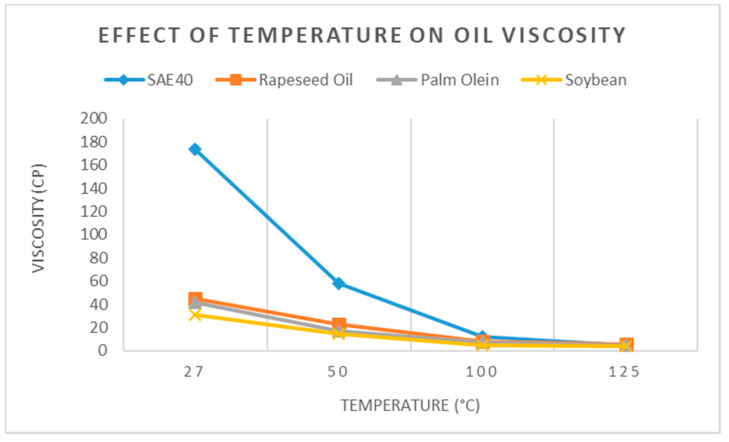
Effect of temperature on oil viscosity.

Figure 5 shows the response of bio-oils and SAE40 against the rising temperature. It can be seen in Figure 5 that the viscosity of SAE40 was relatively higher than that of the three bio-oils at room temperature (27 °C). The sharp decrease in dynamic viscosity was relatively significant for SAE40, from room temperature to 50 °C. Bio-oils exhibited relatively stable dynamic viscosity drop with increasing temperature. This is a significant point in this research, as it provides evidence on the stable dynamic viscosity of bio-oils against rising temperature.

### 3.1. Experimental Modal Analysis

As discussed earlier, the basic purpose to carry out the modal analysis was to verify that there was no natural frequency present in the operating range of the machine. The results show that there was indeed no natural frequency within the operating range.

**Figure 6:**
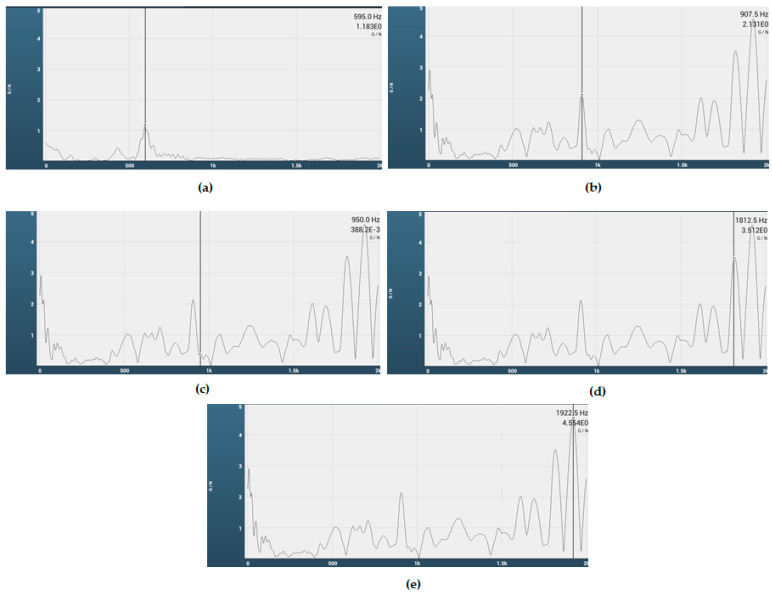
Experimental modal analysis. (**a**) Shows the first mode. (**b**–**e**) Represent the second, third, fourth, and fifth mode, respectively.

Figure 6 shows the experimental modal analysis. Figure 6a shows the first mode, whereas Figure 6b–e represent the second, third, fourth, and fifth mode, respectively. There are a few peaks other than the natural frequencies, which are highlighted and also match with numerical data. This is primarily because, in the numerical data, only a shaft with a disk mounted on it was considered; in the actual setup of JBTR, however, there were several other parts present, which is why there are few other peaks visible in Figure 6, which belong to other components.

### 3.2. Numerical Modal Analysis

**Figure 7:**
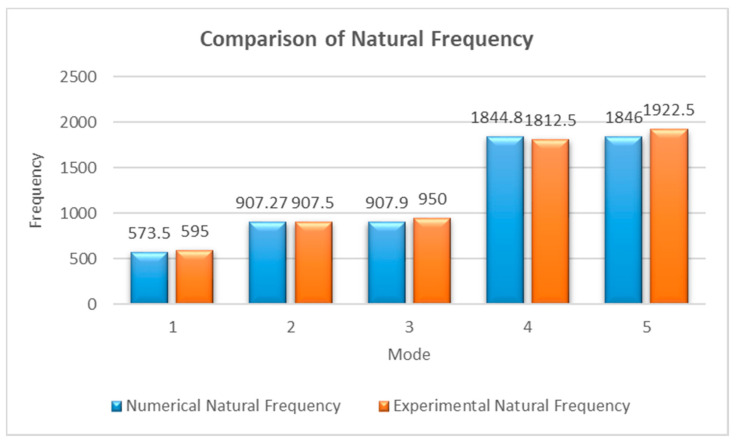
Comparison of numerical and experimental natural frequencies.

For the numerical analysis, the natural frequencies of the shaft and the disk model were analyzed using commercial software ANSYS R2 2021, and the values of the first five natural frequencies are given in Figure 7. Similarly, these values were also experimentally verified, which are given in Figure 7. The experimental and numerical results were relatively similar; the maximum difference observed between the numerical and experimental values was found to be 4.43%, in the third mode. 

In Figure 7, the results of the comparison of experimental and numerical natural frequencies are illustrated. The experimental and numerical data yielded almost the same values for the first five natural frequencies. Although there were several other peaks observed in the experimental data, those peaks may be due to other subparts existing in the system, which were not considered for numerical analysis.

### 3.3. Bearing Temperature Measurement

Once all basic tests and procedures were performed to verify the hypothesis about the static performance of bio-oils, these oils were tested, so as to evaluate their performance in actual operating conditions, the results of which were compared against the SAE40 mineral-based lubricant. The JBTR system was made to operate at different speeds for different types of given oil samples for one hour in each test. The sole purpose of operating the JBTR for one hour was to observe the extended responses of bio-oils under the actual operating and loading conditions.

**Figure 8:**
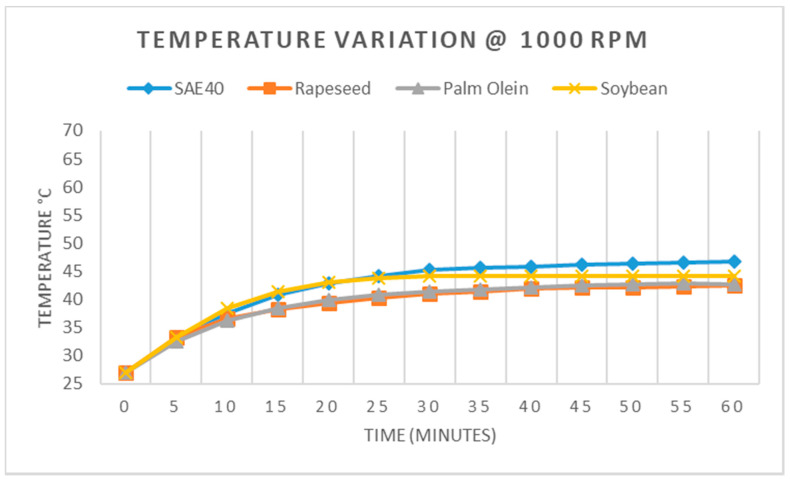
Temperature variation at 1000 RPM.

From Figure 8, it can be seen that the highest temperature rise among the tested oil samples at 1000 rpm was recorded for SAE40 at 46.8 °C after one hour of operation. Soybean oil recorded 44.1 °C after one hour of operation. The response of palm olein and rapeseed oil was relatively similar, with rapeseed oil recording 42.5 °C and palm olein recording 42.7 °C after one hour. In general, the overall trend was rather similar for all the oils having followed the identical path.

**Figure 9:**
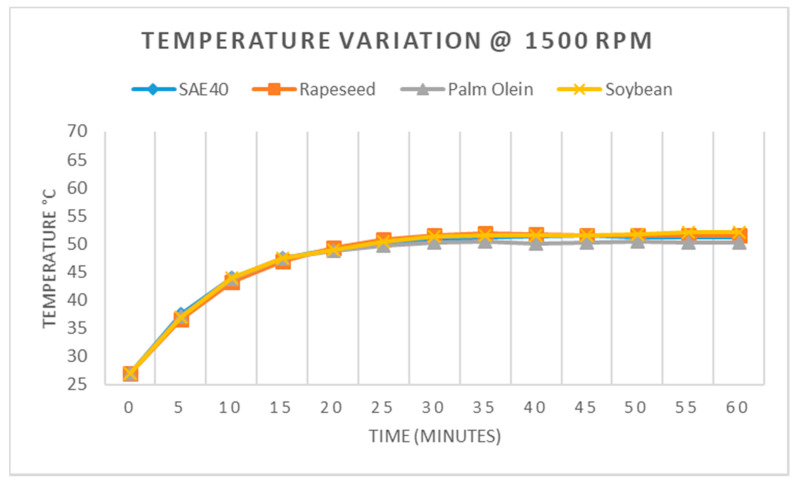
Temperature variation at 1500 RPM.

From Figure 9, it can be seen that the highest temperature rise among the tested oil samples at 1500 rpm was detected for soybean, which recorded 52.1 °C after one hour of operation. Rapeseed oil recorded 51.5 °C after one hour of operation. The response of SAE40 was 51.2 °C, whereas palm olein recorded 50.3 °C after one hour, which was the minimum value.

**Figure 10:**
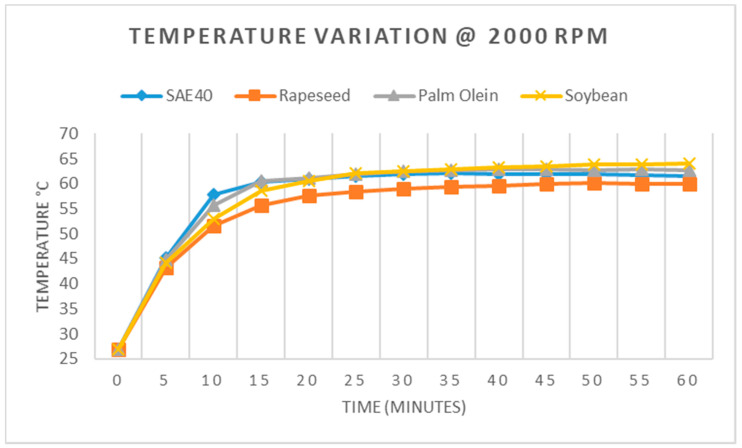
Temperature variation at 2000 RPM.

Similarly, when the machine was made to operate at 2000 rpm, the overall responses were recorded and are shown in Figure 10. The rapeseed oil showed the best response and recorded a temperature of 59.9 °C after one hour of operation. The response of SAE40 was 61.4 °C after one hour, whereas palm olein recorded 62.6 °C, and soybean oil recorded 64 °C after one hour. It is important to mention here that the temperature stabilized after 35 to 40 min of operation, and after that, the rise in temperature was very insignificant (less than 1 °C) for each rotational speed (1000, 1500, 2000 rpm).

**Figure 11:**
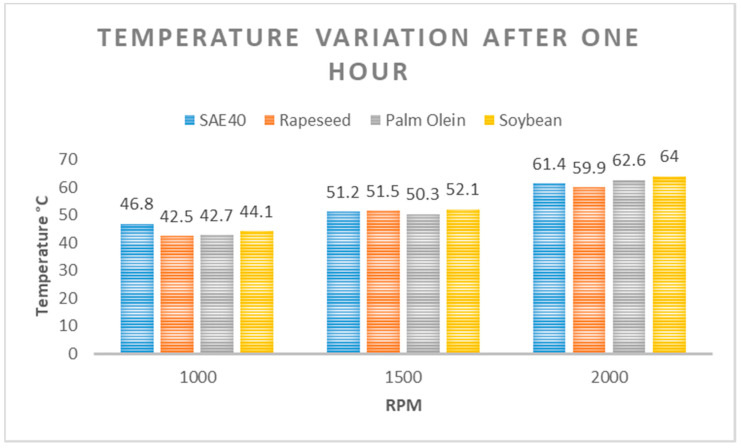
Comparison of temperature at various RPM.

In Figure 11, the overall comparison of responses relative to temperature variation after one hour at three different speeds is provided. The temperature difference was more than 9% for rapeseed oil and SAE40 oil at 1000 rpm after one hour of operation. Although the temperature difference was not very significant after one hour of operation (~2%) for the speed of 1500 and 2000 rpm, it is evident from this comparison that the three bio-oils performed relatively well despite the fact they were used as unprocessed and untreated. This shows the potential of bio-oils for further research. 

## 4. Conclusions

Based on the experimental studies in this research, it is evident that the bio-oils exhibited improved thermal characteristics under the actual operating conditions. The following conclusions can be drawn:

1. For all three speeds, i.e., 1000, 1500, and 2000 rpm, the bio-oils recorded minimum temperature;
2. At 1000 rpm, rapeseed oil recorded a 9.2% lower temperature than SAE40. Similarly, at 2000 rpm, rapeseed oil recorded the minimum temperature, which was 2.5% lower than SAE40;
3. At 1500 rpm, palm olein recorded the minimum temperature, which was 1.8% less than SAE40.

Overall, the results are in line with the literature that bio-oils have good thermal characteristics. Furthermore, the processing of bio-oils can lead to improved characteristics of static and dynamic properties. Similarly, the environmentally friendly nature and biodegradability of bio-oils can be another major factor to accelerate research in this area.

## Figures and Tables

**Table 1:** Specifications of the journal bearing test rig (JBTR).

No.	Description	Specification
1	Bearing length (L)	12.5 mm
2	Inner diameter for plain bearing (d)	25.14 mm
3	Shaft diameter (Φ)	25 mm
4	Weight of journal shaft (W)	9 N
5	Weight of Load	25 N
6	Total clearance (C_T_)	0.14 mm
7	Radial clearance (C_R_)	0.07 mm
8	L/D ratio	0.5
9	Operating speed	1000, 1500, 2000 rpm

**Table 2:** Properties of stainless steel.

Properties	Value
Density	7.75 × 10^−6^ kg mm^−3^
Young’s modulus (MPa)	1.93 × 10^5^
Poisson’s ratio	0.31
Bulk modulus (MPa)	1.693 × 10^5^
Shear modulus (MPa)	73,664

**Table 3:** Physical properties of oil samples.

Properties	SAE40	Palm Olein	Rapeseed	Soya Bean
Flash point (°C)	235	324	326	330
Specific heat, Cp (KJ/Kg-C)	2.53	1.9	1.96	1.88
Thermal conductivity (W/m-C)	0.145	0.172	0.168	0.185
Density at 15 °C (g/cm^3^)	0.890	0.912	0.915	0.924

## Data Availability

Data are available upon request from the corresponding author. These data are not commercially available due to privacy issues.

## References

[1] Rohm H., Jaros D. (2022). Rheological Methods: Instrumentation. Encycl. Dairy Sci..

[2] Wróblewski P. (2021). Analysis of Torque Waveforms in Two-Cylinder Engines for Ultralight Aircraft Propulsion Operating on 0W-8 and 0W-16 Oils at High Thermal Loads Using the Diamond-Like Carbon Composite Coating. SAE Int. J. Engines.

[3] Bair S., Khonsari M., Winer W. (1998). High-pressure rheology of lubricants and limitations of the Reynolds equation. Tribol. Int..

[4] Li H., Wang Y., Zhong N., Chen Y., Yin Z. Study on the performance of journal bearings in different lubricants by CFD and FSI method with thermal effect and cavitation. Proceedings of the 5th International Conference on Mechanical, Materials and Manufacturing (ICMMM 2018).

[5] Nowak P., Kucharska K., Kami M. (2019). Ecological and Health Effects of Lubricant Oils Emitted into the Environment. Int. J. Environ. Res. Public Health.

[6] Nikolakopoulos P.G., Bompos D.A. (2015). Experimental Measurements of Journal Bearing Friction Using Mineral, Synthetic, and Bio-Based Lubricants. Lubricants.

[7] Ambhore P.J., Hemanadth J., Gawande S.H. (2020). Bio-Lubricants for Hydrodynamic Bearings. J. Bio. Tribo-Corros..

[8] Kržan B. (2010). Study on the Tribological Performance of Vegetable Oils. Goriva i Maz..

[9] Reeves C.J., Menezes P.L., Jen T.-C., Lovell M.R. (2015). The influence of fatty acids on tribological and thermal properties of natural oils as sustainable biolubricants. Tribol. Int..

[10] Syahir A.Z., Zulkifli N.W.M., Masjuki H.H., Kalam M.A., Alabdulkarem A., Gulzar M., Khuong L.S., Harith M.H. (2017). A review on bio-based lubricants and their applications. J. Clean. Prod..

[11] Shahabuddin M., Masjuki H., Kalam M., Bhuiya M., Mehat H. (2013). Comparative tribological investigation of bio-lubricant formulated from a non-edible oil source (Jatropha oil). Ind. Crop. Prod..

[12] Zulkifli N., Kalam A., Masjuki H., Shahabuddin M., Yunus R. (2013). Wear prevention characteristics of a palm oil-based TMP (trimethylolpropane) ester as an engine lubricant. Energy.

[13] Wróblewski P., Rogólski R. (2021). Experimental Analysis of the Influence of the Application of TiN, TiAlN, CrN and DLC1 Coatings on the Friction Losses in an Aviation Internal Combustion Engine Intended for the Propulsion of Ultralight Aircraft. Materials.

[14] Trzepieciński T. (2020). Tribological Performance of Environmentally Friendly Bio-Degradable Lubricants Based on a Combination of Boric Acid and Bio-Based Oils. Materials.

[15] Borugadda V.B., Goud V.V. (2016). Improved thermo-oxidative stability of structurally modified waste cooking oil methyl esters for bio-lubricant application. J. Clean. Prod..

[16] Chaurasia S.K., Singh N.K., Singh L.K. (2020). Friction and wear behavior of chemically modified Sal (Shorea Robusta) oil for bio based lubricant application with effect of CuO nanoparticles. Fuel.

[17] Gemsprim M.S., Babu N., Udhayakumar S. (2021). Tribological evaluation of vegetable oil-based lubricant blends. Mater. Today Proc..

[18] Georgescu C., Solea L.C., Deleanu L. (2019). Additivation of vegetal oils for improving tribological characteristics. IOP Conf. Ser. Mater. Sci. Eng..

[19] Sapawe N., Hanafi M.F., Samion S. (2019). The Use of Palm Oil as New Alternative Biolubricant for Improving Anti-Friction and Anti-Wear Properties. Mater. Today Proc..

[20] Shah R., Woydt M., Zhang S. (2021). The Economic and Environmental Significance of Sustainable Lubricants. Lubricants.

[21] Mobarak H., Mohamad E.N., Masjuki H., Kalam A., Al Mahmud K., Habibullah M., Ashraful A. (2014). The prospects of biolubricants as alternatives in automotive applications. Renew. Sustain. Energy Rev..

[22] Zahid R., Bhutta M.U., Mufti R.A., Abdullah M.U., Masjuki H.H., Varman M., Kalam M.A., Ali M.A., Aslam J., Akhtar K. (2021). Friction and Wear Performance Evaluation of Bio-Lubricants and DLC Coatings on Cam/Tappet Interface of Internal Combustion Engines. Materials.

[23] Chatterton S., Pennacchi P., Vania A. (2018). Optimized Tribo-Design of Lubricants for Power Loss Reduction in Journal Bearings Used in Process Industry.

[24] Rao T.V.V.L.N., Rani A.M.A., Awang M., Baharom M. (2018). An overview of research on biolubricants in Malaysia and Japan for tribological applications. J. Tribol..

[25] Cecilia J.A., Ballesteros Plata D., Alves Saboya R.M., Tavares de Luna F.M., Cavalcante C.L., Rodríguez-Castellón E. (2020). An Overview of the Biolubricant Production Process: Challenges and Future Perspectives. Processes.

[26] Mitchell M.R., Link R.E., Wale D., Mba D. (2007). A Journal Bearing Test Rig with Reduced Uncertainty: Some Design Considerations. J. Test. Eval..

[27] Mirev A., Rakanov Y. Hydrodynamic journal bearing test rig with elastic deformations of contact surfaces capabilities. Proceedings of the XV International Scientific Conference “RE & IT–2016” Renewable Energies and Innovative Technologies.

[28] Ciulli E., Forte P., Libraschi M., Nuti M. (2018). Set-up of a novel test plant for high power turbomachinery tilting pad journal bearings. Tribol. Int..

[29] Li Q., Zhang S., Wang Y., Xu W., Wang Z. (2019). A dynamic response test rig of a full-scale rotor—Journal bearing system. Proc. Inst. Mech. Eng. Part J J. Eng. Tribol..

[30] Cloud C.H., Byrne J.M. Fundamentals of Fluid Film Journal Bearing Operation and Modeling. Proceedings of the 34th Turbomachinery Conference.

[31] Childs P.R.N. (2021). Shafts. Mech. Des..

[32] Nik W.W., Ani F.N., Masjuki H., Giap S.E. (2005). Rheology of bio-edible oils according to several rheological models and its potential as hydraulic fluid. Ind. Crop. Prod..

[33] Idrees Ali S., Reshi M. (2018). Rheological review on potential of bio-lubricants. Int. J. Chem. Stud..

